# Indocyanine green fluorescence image-guided total laparoscopic living donor right hepatectomy: The first case report from Mainland China

**DOI:** 10.1016/j.ijscr.2018.11.033

**Published:** 2018-11-22

**Authors:** Xiangfei Meng, Hongguang Wang, Yinzhe Xu, Mingyi Chen, Weidong Duan, Shichun Lu

**Affiliations:** Department of Hepatobiliary Surgery, Chinese PLA General Hospital, 28 Fuxing Road, Haidian, Beijing, 100853, China

**Keywords:** A2ALDLT, adult-to-adult living donor liver transplantation, ICG, indocyanine green, IGFI, indocyanine green fluorescence image, TLDRH, total laparoscopic living donor right hepatectomy, BMI, body mass index, HBV, hepatitis B virus, MELD, model for end-stage liver disease, SLV, standard liver volume, MRCP, magnetic resonance cholangiopancreatography, MHV, middle hepatic vein, GRWR, graft to recipient body weight ratio, RHV, right hepatic vein, IOUS, intraoperative ultrasonography, CUSA, cavitron ultrasonic surgical aspirator, POD, postoperative day, Living donor liver transplantation, Laparoscopy, Indocyanine green, Fluorescence, Right hepatectomy

## Abstract

**Introduction:**

Total laparoscopic living donor right hepatectomy (TLDRH) is sporadically reported worldwide. Liver transection margin used to be determined by ischemic demarcation or intraoperative ultrasonography. To identify the site of bile duct division relied on preoperative MRCP and intraoperative cholangiography, which is experience demanding.

**Presentation of case:**

A 34-year-old man volunteered for living donation to his brother who suffered decompensated HBV-related cirrhosis. Right lobe donation without MHV fulfilled the volumetric criteria. After hilum dissection, ICG was injected into the right portal branch. Right lobe was transected tracing the real-time fluorescence-enhanced borderline and the course of MHV. The right bile duct was transected above the bifurcation that was fluorescently visualized within the parenchyma. The liver graft was retrieved from a pre-made suprapubic incision after simple vascular clamping. The warm ischemia time was 6 min. The recipient procedure was successful with back-table graft venoplasty using cryopreserved iliac artery allografts. The donor recovered uneventfully and was discharged from hospital on POD 7.

**Discussion:**

The operative time, blood loss and postoperative course of donor is comparable to those undergoing ordinary laparoscopic right hepatectomy in our institute. ICG fluorescence can real-timely visualize the surgical margin and biliary branches of right lobe, which helps preserve every last bit of functional liver volume for the donor and avoid the complicated traditional intraoperative cholangiography.

**Conclusion:**

TLDRH proves to be achievable in surgical teams confortable with both laparoscopic hepatectomy and LDLT. ICG fluorescence navigation could make the procedure simplified, safer and more accurate. More practice and technical modification are necessary.

## Highlights

• Total laparoscopic living donor right hepatectomy is rarely reported worldwide.
• Right liver transection plane used to be determined through ischemic demarcation and IOUS.
• The site of bile duct division was determined according to MRCP or intraoperative cholangiography.
• ICG fluorescence can real-timely visualize the surgical margin and biliary branches of right lobe.
• ICG fluorescence navigation makes the procedure simplified, safer and more accurate.

## 1 Introduction

Adult-to-adult living donor liver transplantation (A2ALDLT) has become an established mode of treatment for end-stage liver disease, especially in East Asia [1]. Minimally invasive approach of liver procurement may enhance the donor’s safety and contribute to the acceptability of live donation [2]. With acquired experience in both laparoscopic hepatectomy and graft harvesting in live donors, laparoscopic left lateral sectionectomy has become an accepted manner in pediatric LDLT [3]. But in adults, where the right lobe donation is mostly preferred, laparoscopy raises concerns about not only graft integrity but also donor safety [4]. This is likely why, until now, the pure laparoscopic retrieval of the right lobe was only sporadically reported in some pioneering teams [2,4,5].

Biliary variation commonly occurred in the right lobe and, to ensure donor safety and graft integrity, intraoperative cholangiography is often mandatory to confirm the configuration of biliary tree. It is difficult to replicate the maneuver of biliary intubation and radiography under laparoscope and failure to recognize bile duct anatomy might cause conversion to laparotomy. Moreover, the preoperative MRCP and intraoperative C-arm X-ray images differ from the laparoscopic caudal-to-cranial view, thusly compromising its navigating value.

Indocyanine green (ICG) fluorescence imaging is a novel technique applied in hepatobiliary surgery [6,7]. With an infrared fluorescence probe mounted to the laparoscopy system, the portal territory and biliary tree can be visualized in a real-time and three-dimensional manner [8]. This could help surgeons to transect the right lobe tracing its anatomical margin and accurately identify the biliary tract, which will enhance the donor safety by preserving every last bit of functional liver volume and the structural integrity of remnant liver. We hereby report the first case of ICG fluorescence image (IGFI)-guided total laparoscopic living donor right hepatectomy (TLDRH) in Mainland China. The work has been reported in line with the SCARE criteria [9] and the PROCESS criteria [10].

## 2 Case report

**Fig. 1:**
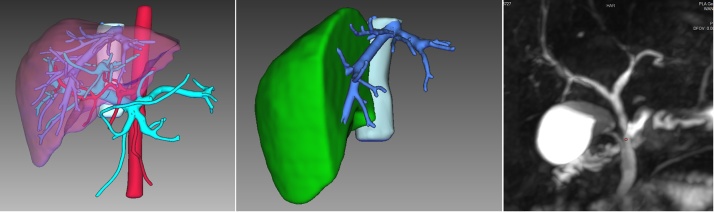
3D image reconstruction and MRCP of the donor liver.

A 30-year-old male (170 cm/60 kg, BMI = 20.8) suffered the HBV-related cirrhosis complicated by portal hypertension. His model for end-stage liver disease (MELD) score was 19. His liver anatomy was modal with a standard liver volume (SLV) of 1220cm^3^ [11]. Transplantation was considered due to his deteriorated general and liver condition. His 34-year-old brother (170 cm/70 kg, BMI = 23.9) volunteered for living donation. He underwent a comprehensive living donor evaluation including MRCP and the CT image-based three-dimensional reconstruction (IQQA-liver, EDDA, US) for volumetric assessment and angiography. The volume of his right lobe (i.e. segments 5–8) without middle hepatic vein (MHV) was 672cm^3^, representing 54.3% of his entire liver. The graft to recipient body weight ratio (GRWR) was 1.32% and the liver remnant was 45.7% of the total liver volume, which fulfills the safety criteria for both recipient and donor. Donor’s hepatic vasculature anatomy was modal with the presence of portal vein bifurcation and a single right branch of a proper hepatic artery. MRCP showed normal biliary confluence with a single right hepatic duct. MHV tributaries in Segment 5, Segment 8 ventral and dorsal areas were ＞5 mm in diameter and need to be reconstructed for the recipient Fig. 1. Informed consent was obtained from both donor and recipient, which were characterized by a morbi-mortality of this complex procedure and the innovative nature of the pure laparoscopic technique. It’s decided that any incident occurring during the innovative procedure that might compromise the donor safety or graft integrity would prompt conversion to laparotomy. The procedure was approved by the ethics committee of our institute.

**Fig. 2:**
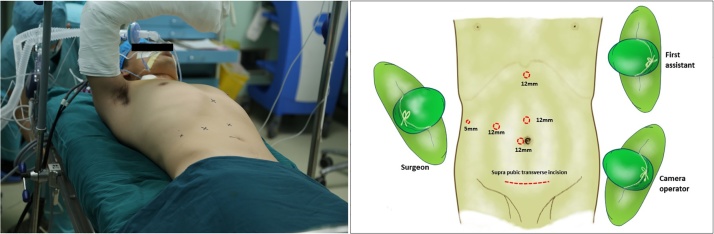
Patient posture and trocar placement.

**Fig. 3:**
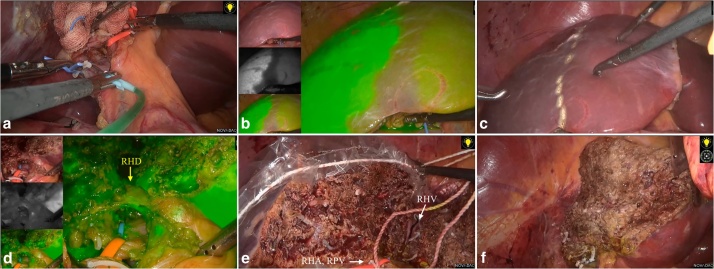
Intraoperative images. a. ICG injection into the right portal vein; b. Fluorescence mapping of the right lobe; c. Ischemic demarcation line of the right lobe; d. ICG fluorescent visualization of the right hepatic duct; e. Right lobe in retrieval bag with right hepatic artery (RHA), right portal vein (RPV) and right hepatic vein (RHV) isolated; f. Remnant liver.

**Fig. 4:**
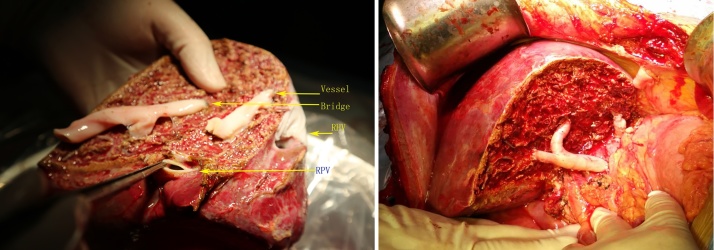
Back-table graft venoplasty and liver implantation.

A surgical team (XM, HW, YX) with an experience of over 500 cases of laparoscopic major hepatectomy performed the donor procedure. The donor was placed in a supine position with right upper abdomen slightly elevated and right arm suspended. Four trocars were placed as shown in Fig. 2. A pneumoperitoneum was created and maintained at 13 mmHg. The cystic duct and artery were clipped and divided. The portal pedicle was opened with the right hepatic artery and right portal branch dissected free and taped, during which some small portal branches into the caudate lobe were suture-ligated and divided. After cutting the round and falciform ligaments, the right lobe was mobilized with sectioning of the short hepatic veins at anterior aspect of the inferior vena cava. The right hepatic vein (RHV) was then dissected and taped. 2 ml of ICG (0.5 mg/ml) was directly injected into the right portal branch through a scalp needle. The fluorescent territory of the right lobe was clearly visualized through the fusion image mounted on the laparoscopy system (Pinpoint, Stryker, US). The right hepatic artery and portal branch were briefly clamped to reveal the main portal fissure and to confirm the transection plane Fig. 3**a**–**c**. The intra-operative ultrasound (IOUS) was used to identify the course of MHV, which would be preserved for the left lobe. The liver capsule was opened using a harmonic scalpel. Parenchyma was divided tracing the fluorescence borderline of right lobe. A Cavitron Ultrasonic Surgical Aspirator (CUSA) was used for parenchyma dissection. Bipolar coagulation and clips were used for hemostasis. The Segment 5, 8 ventral and dorsal venous tributaries were double clipped and divided. When the right hepatic duct level was reached, 20 ml of ICG was bolus injected intravenously. The biliary confluence and right hepatic duct were then clearly visualized and sectioned. The liver section was then completed and the right hepatic vein was isolated. A 12-cm suprapubic incision without muscle sectioning was made allowing for the insertion of a retrieval bag. The right lobe was put into the bag before the right hepatic artery, portal branches and hepatic vein were a securely clipped and divided Fig. 3**d**–**f**. The graft was quickly retrieved to the back table and perfused with cold UW solution. Then the right hepatic ducal stump on the liver remnant was sealed by suture. The warm ischemia time was 6 min 20 s. The V5, V8v and V8d were recanalized on the back-table using cryopreserved iliac artery allografts Fig. 4. The recipient procedure and perioperative management followed our institutional protocol. The donor procedure took 420 min with a blood loss of 100 ml. The recipient procedure took 380 min with a blood loss of 450 ml. Neither required blood transfusion. The donor recovered uneventfully and was discharged from hospital on POD 7.

## 3 Discussion

Laparoscopic living donation has become a gold-standard technique for adult renal transplantation, with reduced morbidity and faster recovery and therefore an increased donation rate [12]. Due to the technical complexity and anatomical variation, total laparoscopic living donor liver procurement remains challenging, especially in right lobe donation [4,5]. To ensure donor’s safety, the Pringle maneuver is abolished and surgeons must face the greater intraoperative blood loss and the necessity of transfusion. And to confirm the remnant bile duct, intraoperative cholangiography is often mandatory. Its maneuver applied in open surgery may not be replicated under microscope. And the coronary image based on MRCP or C-arm X-ray images differ from the laparoscopic caudal-to-cranial view, which might compromise their navigating value. Bleeding, failure to recognize biliary anatomy, vessel injury and failure to progress can all result in conversion to laparotomy [2,4]. Moreover, in contrast to oncological lobectomy, to ensure the graft integrity, the right lobe has to be meticulous sectioned without vascular deprivation and with adequate vessel stump for recanalization. In a word, TLDRH is technically demanding for surgical precision [13].

Our initial experiences from this case are: (1) This procedure should be limited to surgical teams that are experienced in both laparoscopic major hepatectomy and living donor liver harvesting techniques; (2) Exhaustive living donor evaluation, including the volumetric, vascular and biliary analysis, is of paramount importance. Three-dimensional image reconstruction is mandatory to visualize the anatomical configuration and to simulate the laparoscopic surgical view; (3) A meticulous fashion of liver transection should be applied to prevent bulky sealing of the graft vasculature and intraoperative bleeding; (4) Real-time fusion ICG fluorescence imaging technique adds much precision to the liver transection and bile duct identification.

ICG fluorescence imaging technique has been rapidly expanding in the field of hepatobiliary surgery [7,14,15]. Guided by real-time fusion ICG fluorescence image, surgeons do not have to rely on their guesswork of inter-segment plane or frequently try IOUS to trace the landmark vessels [14]. Tracing the portal territory borderline of right lobe can help preserve every last bit of functional liver remnant for the donor. Moreover, due to the specifically liver ingestion and biliary secretion, ICG fluorescence can also help identify the biliary bifurcation and right hepatic duct. With a fusion image mounted on the laparoscopy system, surgeons do not have to avert their gaze between the MRCP or intraoperative cholangiography and the laparoscopic view. Some lessons have also been learned from our initial practice. Spillover of ICG may occur during the portal vein injection in our case. Thus we suggest suspending the portal branch to prevent spillover and injecting ICG more slowly. The procedure should be restricted to highly selected cases, especially those with modal liver anatomical pattern.

## 4 Conclusion

TLDRH proves to be achievable in surgical teams confortable with both laparoscopic hepatectomy and LDLT. ICG fluorescence navigation bears the potential to make the procedure simplified, safer and more accurate. More practice and technical modification are necessary.

## Conflicts of interest

Drs. Xiangfei Meng, Hongguang Wang, Yinzhe Xu, Mingyi Chen, Weidong Duan, Shichun Lu have no conflicts of interest or financial tie to disclose as described by the Journal.

## Funding

The work has no grants or financial support.

## Ethical approval

The procedure was approved by the Ethics Committee at the Chinese PLA General Hospital.

## Consent

Written informed consent was obtained from the patient for publication of this case report and any accompanying images.

## Author contribution

Drs. Xiangfei Meng, Hongguang Wang, Yinzhe Xu and Mingyi Chen performed the donor procedure, drafted, proofread and edited the manuscript.

Drs. Yinzhe Xu, Weidong Duan and Shichun Lu performed the recipient procedure.

Dr. Shichun Lu conceptualized the idea and has given the final approval of the version to be published.

## Registration of research studies

This is not the first-in-man study worldwide. It is our initial practice in Mainland China with many innovative experience. The patient data has been registered in the national patient database and the hospital data base.

## Guarantor

Dr. Shichun Lu.

## Provenance and peer review

Not commissioned, externally peer reviewed.

## References

[1] Chen C.L., Kabiling C.S., Concejero A.M. (2013). Why does living donor liver transplantation flourish in Asia?. Nat. Rev. Gastroenterol. Hepatol..

[2] Suh K.S., Hong S.K., Lee K.W., Yi N.J., Kim H.S., Ahn S.W., Yoon K.C., Choi J.Y., Oh D., Kim H. (2018). Pure laparoscopic living donor hepatectomy: focus on 55 donors undergoing right hepatectomy. Am. J. Transplant..

[3] Soubrane O., Cherqui D., Scatton O., Stenard F., Bernard D., Branchereau S., Martelli H., Gauthier F. (2006). Laparoscopic left lateral sectionectomy in living donors: safety and reproducibility of the technique in a single center. Ann. Surg..

[4] Soubrane O., Perdigao Cotta F., Scatton O. (2013). Pure laparoscopic right hepatectomy in a living donor. Am. J. Transplant..

[5] Lee K.W., Hong S.K., Suh K.S., Kim H.S., Ahn S.W., Yoon K.C., Lee J.M., Cho J.H., Kim H., Yi N.J. (2018). One hundred and fifteen cases of pure laparoscopic living donor right hepatectomy at a single center. Transplantation.

[6] Kobayashi Y., Kawaguchi Y., Kobayashi K., Mori K., Arita J., Sakamoto Y., Hasegawa K., Kokudo N. (2017). Portal vein territory identification using indocyanine green fluorescence imaging: technical details and short-term outcomes. J. Surg. Oncol..

[7] Kawaguchi Y., Nomura Y., Nagai M., Koike D., Sakuraoka Y., Ishida T., Ishizawa T., Kokudo N., Tanaka N. (2017). Liver transection using indocyanine green fluorescence imaging and hepatic vein clamping. Br. J. Surg..

[8] Terasawa M., Ishizawa T., Mise Y., Inoue Y., Ito H., Takahashi Y., Saiura A. (2017). Applications of fusion-fluorescence imaging using indocyanine green in laparoscopic hepatectomy. Surg. Endosc..

[9] Agha R.A., Fowler A.J., Saeta A., Barai I., Rajmohan S., Orgill D.P., Group S. (2016). The SCARE statement: consensus-based surgical case report guidelines. Int. J. Surg..

[10] Agha R.A., Fowler A.J., Rajmohan S., Barai I., Orgill D.P., Group P. (2016). Preferred reporting of case series in surgery; the PROCESS guidelines. Int. J. Surg..

[11] Urata K., Kawasaki S., Matsunami H., Hashikura Y., Ikegami T., Ishizone S., Momose Y., Komiyama A., Makuuchi M. (1995). Calculation of child and adult standard liver volume for liver transplantation. Hepatology.

[12] Schweitzer E.J., Wilson J., Jacobs S., Machan C.H., Philosophe B., Farney A., Colonna J., Jarrell B.E., Bartlett S.T. (2000). Increased rates of donation with laparoscopic donor nephrectomy. Ann. Surg..

[13] Dong J., Yang S., Zeng J., Cai S., Ji W., Duan W., Zhang A., Ren W., Xu Y., Tan J., Bu X., Zhang N., Wang X., Wang X., Meng X., Jiang K., Gu W., Huang Z. (2013). Precision in liver surgery. Semin. Liver Dis..

[14] Aoki T., Yasuda D., Shimizu Y., Odaira M., Niiya T., Kusano T., Mitamura K., Hayashi K., Murai N., Koizumi T., Kato H., Enami Y., Miwa M., Kusano M. (2008). Image-guided liver mapping using fluorescence navigation system with indocyanine green for anatomical hepatic resection. World J. Surg..

[15] Ishizawa T., Saiura A., Kokudo N. (2016). Clinical application of indocyanine green-fluorescence imaging during hepatectomy. Hepatobiliary Surg. Nutr..

